# Metrics of High-Density Lipoprotein Function and Hospital Mortality in Acute Heart Failure Patients

**DOI:** 10.1371/journal.pone.0157507

**Published:** 2016-06-15

**Authors:** Ines Potočnjak, Vesna Degoricija, Matias Trbušić, Sanda Dokoza Terešak, Bojana Radulović, Gudrun Pregartner, Andrea Berghold, Beate Tiran, Gunther Marsche, Saša Frank

**Affiliations:** 1 University Hospital Centre Sisters of Charity, Department of Medicine, Zagreb, Croatia; 2 University of Zagreb School of Medicine, Zagreb, Croatia; 3 University Hospital Centre Zagreb, Zagreb, Croatia; 4 Institute for Medical Informatics, Statistics und Documentation, Medical University of Graz, Graz, Austria; 5 Clinical Institute for Medical and Chemical Laboratory Diagnostics, Medical University of Graz, Graz, Austria; 6 Institute of Experimental and Clinical Pharmacology, Medical University of Graz, Graz, Austria; 7 Institute of Molecular Biology and Biochemistry, Centre of Molecular Medicine, Medical University Graz, Graz, Austria; University of Milano, ITALY

## Abstract

**Objective:**

The functionality of high-density lipoprotein (HDL) is impaired in chronic ischaemic heart failure (HF). However, the relationship between HDL functionality and outcomes in acute HF (AHF) has not been studied. The present study investigates whether the metrics of HDL functionality, including HDL cholesterol efflux capacity and HDL-associated paraoxonase (PON)-1 arylesterase (AE) activity are associated with hospital mortality in AHF patients.

**Methods and Results:**

The study was performed as a prospective, single-centre, observational research on 152 patients, defined and categorised according to the ESC and ACCF/AHA Guidelines for HF by time of onset, final clinical presentation and ejection fraction. The mean age of the included patients (52% female) was 75.2 years (SD 10.3) and hospital mortality was 14.5%. HDL cholesterol efflux capacity was examined by measuring the capacity of apoB depleted serum to remove tritium-labelled cholesterol from cultured macrophages. The AE activity of the HDL fraction was examined by a photometric assay. In a univariable regression analysis, low cholesterol efflux, but not AE activity, was significantly associated with hospital mortality [odds ratio (OR) 0.78, 95% confidence interval (CI) 0.64–0.96, p = 0.019]. In multivariable analysis progressively adjusting for important clinical and laboratory parameters the association obtained for cholesterol efflux capacity and hospital mortality by univariable analysis, despite a stable OR, did not stay significant (p = 0.179).

**Conclusion:**

Our results suggest that HDL cholesterol efflux capacity (but not AE activity) contributes to, but is not an independent risk factor for, hospital mortality in AHF patients. Larger studies are needed to draw firm conclusions.

## Introduction

Clinical and epidemiological studies demonstrated an inverse relationship between high-density lipoprotein cholesterol (HDL-cholesterol) levels and cardiovascular disease (CVD) [1]. However, recent studies provided evidence against HDL-cholesterol as a potential therapeutic target. Along these lines, a study using the Mendelian randomisation approach failed to reveal an association between genetic variants that raise HDL-cholesterol plasma concentrations and a lower risk of cardiovasuclar events [2]. Furthermore, pharmacological interventions aimed at raising HDL-cholesterol levels failed to reduce cardiovascular events [3, 4]. Determination of HDL particle concentrations and HDL subclasses as well as determination of HDL functionality have proved more appropriate to test and quantify the beneficial atheroprotective properties of HDL [5, 6]. HDL-mediated atheroprotection has been ascribed to the attenuation of endothelial adhesion molecule expression, protection of low-density lipoprotein (LDL) from oxidation, inhibiton of inflammatory response in macrophages, stimulation of endothelial nitric oxide production and promotion of vasodilatation [7–11]. The best-studied protective activity of HDL is the promotion of reverse cholesterol transport, a dynamic process by which HDL removes cholesterol from the periphery for delivery back to the liver for excretion [12]. Recent studies provided strong evidence that HDL-cholesterol efflux capacity, the first step in the process of reverse cholesterol transport, is inversely associated with incident coronary heart events, independent of established cardiovascular risk factors [13].

HDL-associated paraoxonase-1 (PON-1) emerged as an important mediator of many protective functions of HDL [7, 14, 15]. In line with this, PON-1 knockout mice show an enhanced susceptibility for developing atherosclerosis [16], whereas PON-1 overexpressing mice are protected from the development of atherosclerosis and exhibit reduced systemic measures of oxidation [17]. Reduced systemic PON-1 activity in humans, as monitored by serum arylesterase (AE) activity levels, was found to be accompanied by increased systemic oxidative stress and to predict an increased risk for major adverse cardiac events [18].

Heart failure (HF) can be defined as an abnormality of cardiac structure or function leading to failure of the heart to deliver oxygen at a rate commensurate with the requirements of the metabolising tissues, despite normal filling pressures (or only at the expense of increased filling pressures) [19, 20]. Acute heart failure (AHF) is the term used to describe the rapid onset of, or change in, symptoms and signs of HF [20]. Previous studies showed that low HDL-C levels were associated with increased mortality and adverse prognosis in HF patients [21, 22]. Importantly, both the antioxidative and cholesterol efflux capacities of HDL were found to be reduced in HF patients when compared with healthy controls [18, 23].

In the present study we examined whether HDL-cholesterol efflux capacity and HDL-associated PON-1 AE activity are risk factors for hospital mortality in AHF patients. To get an integrated measure of HDL quantity and quality, we assessed the metrics of HDL functionality in apoB-depleted serum. We found that HDL-cholesterol efflux capacity (but not AE activity), contributes to, but is not an independent risk factor for hospital mortality in AHF patients.

## Materials and Methods

### Study design and Patients

The AHF study was performed as a prospective, single-centre, observational research study including consecutive adult white hospitalised AHF patients. The study was approved by the local Ethics Committee of the University Hospital Centre Sisters of Charity, Zagreb, Croatia (UHC SC) as well as by the local Ethics Committee of the Medical University of Graz. Written informed consent was obtained from each patient in compliance with Good Clinical Practice, and the investigation conforms with the principles outlined in the *Declaration of Helsinki* [24]. In total, 152 patients were recruited from the Emergency Department from November 2013 to February 2015. The patients were defined and categorised according to the ESC and ACCF/AHA Guidelines for HF by time of onset, final clinical presentation and ejection fraction (EF) [19, 20, 25]. All patients were treated in accordance with standard ESC Guidelines for AHF [20, 25].

### Laboratory assays

Blood samples for routine laboratory assays were obtained from the AHF patients at admission to the hospital. The blood was collected in 6 mL tubes, VACUETTE^®^ Z Serum Clot Activator (Greiner Bio-one GmbH, Kremsmuenster, Austria) with a special coating on the internal wall of the tubes containing microscopic silica particles to prevent clot formation by surface activation. Serum creatinine, urea, total plasma cholesterol, LDL cholesterol, HDL-cholesterol and triglycerides were measured using a Beckman Coulter instrument AU 2700, 2007 (Brea, CA, SAD) and Architect c8000, Abbott 2013 (Chicago, IL, SAD). Glomerular filtration rate (GFR) was calculated as described [26]. The serum aliquots were stored and transported at -80°C.

### ApoB-depletion of serum

ApoB-depleted serum was prepared by the addition of 40 μL polyethylene glycol (20% in 200 mmol/L glycine buffer) to 100 μL serum. The samples were incubated at room temperature for 20 minutes and the supernatant was recovered after centrifugation (10.000 rpm, 20 minutes, 4°C) as described [27].

### Measurements of HDL cholesterol efflux capacity

HDL cholesterol efflux capacity was quantified using a validated ex vivo assay that measures the ability of apolipoprotein-B depleted serum to mobilize cholesterol from cholesterol-laden macrophages [23, 28–31]. J774 cells, a mouse macrophage cell line [32], were plated and labeled for 24 hours with 1 μCi/mL ^3^H-cholesterol (Perkin Elmer, Boston, MA, USA). J774 cells express very low levels of ATP-binding cassette transporter A1 (ABCA1), an important pathway of cholesterol efflux from macrophages. To upregulate ABCA1, cells were stimulated for 6 hours with serum-free DMEM containing 0.3 mmol/L 8-(4-chlorophenylthio)-cyclic AMP (Sigma, Darmstadt, Germany). After this incubation period, cells were washed and ^3^H-cholesterol efflux was determined by incubating cells with 2.8% apoB-depleted serum for 4 hours as described [28]. Liquid scintillation counting was used to quantify the efflux of radioactive cholesterol from the cells. The quantity of ^3^H-cholesterol incorporated into cellular lipids was assessed by means of isopropanol extraction of ^3^H-cholesterol content of J774 cells not exposed to the patients' serum. The percent efflux was calculated by the following formula: [(microcuries of ^3^H-cholesterol in media containing 2.8% apolipoprotein B–depleted serum—microcuries of ^3^H-cholesterol in serum-free mediums) ÷ microcuries of ^3^H-cholesterol in cells before the efflux step] × 100. All steps were performed in the presence of 2 μg/mL of the acyl coenzyme A cholesterol acyltransferase inhibitor Sandoz 58–035 (Sigma, Darmstadt, Germany). The values shown represent the means of two independent assays performed in duplicates. To correct for interassay variation across plates, we included a serum control on each plate, and we normalised the values for patient serum samples to this value in the subsequent analyses. The intra-assay coefficient of variation was 7.1% and the interassay coefficient of variation was 6.8%.

### AE activity assay

The Ca^2+-^dependent AE activity in apoB-depleted serum was determined using a photometric assay with phenylacetate as the substrate, as previously described [33].

### Statistical analysis

Categorical data are presented as absolute and relative frequencies, continuous data are presented as mean and SD or as median and range (minimum to maximum) according to distribution. The Pearson correlation coefficients were calculated to assess the correlation of AE activity and cholesterol efflux capacity with the clinical and laboratory parameters. To study the impact of AE activity and cholesterol efflux capacity on hospital mortality, a univariable logistic regression analysis was performed. In addition, the impact of the following variables on hospital mortality was analysed: age, gender, body mass index (BMI), hypertension, diabetes mellitus type 2, mean arterial pressure (MAP), New York Heart Association Functional Classification (NYHA) classes, ejection fraction (EF), high-density lipoprotein (HDL) cholesterol, low-density lipoprotein (LDL) cholesterol, total cholesterol, triglycerides (logarithmic scale) and GFR. To further explore the relationship of the cholesterol efflux capacity and hospital mortality, we progressively adjusted for important clinical and laboratory parameters. All variables that were associated with hospital mortality in the univariable analysis (p ≤ 0.02) were included as potential covariates in a multiple logistic regression model [34]. Forward selection with a p enter < 0.15 was used and the multicollinearity among covariates was assessed between these variables using the variance inflation factors (VIF > 4). Goodness of fit was checked using the Hosmer and Lemeshow χ2 statistics (p **>** 0.05). All data were analysed using R version 3.2.2.

## Results

### Patients

In this study 152 patients were included, 79 (52%) were female. The mean age of the included patients was 75.2 years (SD 10.3) and the mean BMI was 28.8 kg/m^2^ (SD 5.4). Worsening of chronic HF was present in the majority (69.1%) of the studied patients. Recorded frequent comorbidities were hypertension (89.5%), metabolic syndrome (55.9%), type 2 diabetes mellitus (51.7%), hyperlipidaemia/hypertriglyceridaemia (39.5%) and hypercholesterolaemia (38.8%). Hospital mortality was 14.5%. The patients' baseline characteristics are presented in Table 1.

**Table 1:** Baseline characteristics, biochemical laboratory parameters, comorbidities, and classification of AHF patients (n = 152).

**Baseline characteristics**	
Age (years)	75.2 (10.3)
Gender Female	79 (52%)
Body Weight (kg)	82.9 (19.9)
BMI (kg/m^2^)	28.8 (5.4)
Waist Circumference (cm)	111.2 (17.7)
MAP (mmHg)	105.4 (21.9)
EF (>40%)*	74 (52.1%)
NYHA class 2	11 (7.2%)
NYHA class 3	83 (54.6%)
NYHA class 4	58 (38.2%)
**Biochemical laboratory parameters**	
Triglycerides (mmol/L) ^a^	1.1 [0.5–4.3]
Total Cholesterol (mmol/L) ^a^	3.8 [1.7–9.1]
HDL-cholesterol (mmol/L)^a^	1.0 [0.3–3.6]
LDL-cholesterol (mmol/L) ^a^	2.3 [0.8–6.3]
GFR (ml/min/1.73 m^2^)	50.9 [15.0–105.7]
**Comorbidities**	
Hypertension	136 (89.5%)
Type 2 Diabetes Mellitus	78 (51.7%)
Hyperlipidaemia	60 (39.5%)
Hypercholesterolemia	59 (38.8%)
**Classification of acute heart failure by time of onset**	
Worsening of Chronic Heart Failure	105 (69.1%)
De novo	47 (30.9%)
**Final Clinical Presentation**	
Worsening of Chronic Heart Failure	78 (51.3%)
Acute Coronary Syndrome and HF	23 (15.1%)
Hypertensive AHF	22 (14.5%)
Pulmonary Edema	20 (13.2%)
Isolated Right Side AHF	7 (4.6%)
Cardiogenic Shock	2 (1.3%)
Classification of acute heart failure by Ejection Fraction**	
HFrEF	83 (57.6%)
HFpEF	61 (42.4%)

Metric, continuous, quantitative variables are presented as mean and standard deviation if normally distributed and median with interquartile range if skewed. Categorical, qualitative variables are presented as the absolute and relative frequencies are given as percentage. AHF, Acute Heart Failure; HDL, High-Density Lipoprotein; HFpEF, Heart Failure with preserved Ejection Fraction; HFrEF, Heart Failure with reduced Ejection Fraction; LDL, Low-Density Lipoprotein; NYHA, New York Heart Association Functional Classification; GFR, glomerular filtration rate; BMI, Body Mass Index; EF, Ejection Fraction; MAP, Mean Arterial Pressure

^a^SI measurements. To convert to mg/dL, multiply TG by 89 and TC, HDL, or LDL by 39.

*Based on measurement for 142 patients

**Based on measurement for 144 patients

### Correlation of HDL cholesterol efflux and HDL-associated PON-1 AE activity with clinical and laboratory variables

As shown in Table 2, both HDL cholesterol efflux capacity and AE activity were correlated with total cholesterol, HDL-cholesterol and LDL-cholesterol, as well as with MAP. Furthermore, AE activity correlated with log(triglycerides) and GFR. Neither efflux nor AE activity were correlated with BMI or EF. The AE activity and cholesterol efflux capacity were significantly correlated with each other (r = 0.43; p < 0.001).

**Table 2:** Pearson correlation of clinical and laboratory variables with HDL AE activity and cholesterol efflux capacity.

	AE activity (μmol/L/min)		Cholesterol efflux (%)	
	coefficient	p-value	coefficient	p-value
MAP (mmHg)	0.204	**0.013**	0.186	**0.024**
BMI (kg/m^2^)	0.039	0.637	-0.024	0.769
EF (%)	-0.030	0.723	-0.132	0.123
HDL-cholesterol (mmol/L)	0.397	**<0.001**	0.448	**<0.001**
LDL-cholesterol (mmol/L)	0.461	**<0.001**	0.194	**0.018**
Total cholesterol (mmol/L)	0.570	**<0.001**	0.318	**<0.001**
Log(Triglycerides) (mmol/L)	0.328	**<0.001**	0.154	0.061
GFR (ml/min/1.73 m^2^)	0.177	**0.032**	-0.009	0.909

MAP, Mean arterial pressure; BMI, body mass index; EF, ejection fraction; LDL, low-density lipoprotein cholesterol; HDL, high-density lipoprotein; AE, arylesterase;

### Logistic regression analyses

Univariable analyses showed a significant inverse association of cholesterol efflux capacity, but not of AE activity, with hospital mortality. A significant inverse association with hospital mortality was also observed for LDL-cholesterol and total cholesterol as well as for BMI and MAP (Table 3). As expected, higher NYHA class was associated with increased hospital mortality (Table 3). In contrast, no significant associations with hospital mortality were found for HDL-cholesterol, log(triglycerides), age, gender, hypertension, diabetes mellitus type 2, EF and GFR (Table 3).

**Table 3:** Univariable logistic regression analysis of HDL function, clinical and laboratory parameters and hospital mortality.

Variable	Category	N	OR	LB	UB	p-value
**Metrics of HDL function**						
AE (μmol/L/min)			0.995	0.990	1.000	0.069
Cholesterol efflux (%)			0.783	0.639	0.960	**0.019**
**Clinical parameters**						
Age (years)			1.034	0.984	1.09	0.184
Gender	male	8/73	Ref.			
	female	14/79	1.75	0.69	4.45	0.240
BMI (kg/m^2^)			0.91	0.82	1.00	**0.044**
Hypertension	no	3/16	Ref.			
	yes	19/136	0.70	0.18	2.703	0.609
Type 2 Diabetes Mellitus	no	13/73	Ref.			
	yes	9/78	0.60	0.24	1.51	0.278
MAP (mmHg)			0.95	0.93	0.98	**0.001**
NYHA class	2 and 3	7/94	Ref			
	4	15/58	4.34	1.65	11.42	**0.003**
EF (%) *			1.00	0.96	1.04	0.861
**Laboratory parameters**						
HDL-cholesterol (mmol/L)			0.38	0.09	1.6	0.186
LDL-cholesterol (mmol/L)			0.54	0.31	0.96	**0.035**
Total cholesterol (mmol/L)			0.58	0.36	0.91	**0.018**
Log(Triglycerides) (mmol/L)			0.40	0.12	1.42	0.157
GFR (ml/min/1.73 m^2^)			0.99	0.97	1.01	0.437

N, number of events / total number of patients in category; OR, odds ratio; LB, lower bound of 95% confidence interval; UB, upper bound of 95% confidence interval; Ref., reference category; LDL, low-density lipoprotein; HDL, high-density lipoprotein;

*Based on measurment for 142 patients.

The multivariable model for hospital mortality is shown in Table 4. The model predictors of hospital mortality included MAP, NYHA class, LDL-cholesterol and BMI. Although cholesterol efflux was associated with hospital mortality in univariable analysis, there was no significant effect after adjusting for the other factors in the multivariable model (p = 0.179).

**Table 4:** Multivariable logistic regression analysis of hospital mortality.

	OR	LB	UB	p-value
MAP (mmHg)	0.96	0.93	0.99	**0.008**
NYHA class = 4	4.93	1.67	14.49	**0.004**
LDL-cholesterol (mmol/L)	0.54	0.30	1.00	0.051
BMI (kg/m^2^)	0.91	0.82	1.01	0.089

Hosmer-Lemeshow goodness of fit test: p = 0.554, OR, odds ratio; LB, lower bound of 95% confidence interval; UB, upper bound of 95% confidence interval; MAP, Mean arterial pressure; NYHA, New York Heart Association Functional Classification; LDL, low-density lipoprotein; BMI, body mass index;

## Discussion

In the present study we prospectively examined whether metrics of HDL functionality, namely, HDL cholesterol efflux capacity and HDL-associated PON-1 AE activity, were associated with hospital mortality in AHF patients. We found an association between low HDL cholesterol efflux capacity and an increased risk for hospital mortality in AHF patients. A previous study demonstrated a strong association between low serum AE activity and poor long-term prognosis in decompensated systolic HF patients [18]. The lack of impact of AE activity on hospital mortality in the present study might indicate that decreased AE activity is useful only as a long-term prognostic marker of adverse cardiac events.

In line with decreased blood pressure, an established risk for hospital mortality [35], a decreased MAP emerged as an independent predictor of hospital mortality in our AHF cohort. This also fits with ‘‘reverse epidemiology” which describes the paradoxical association of decreased BMI, serum total cholesterol levels and blood pressure, respectively, with increased morbidity and mortality in HF patients [36]. However, high levels of serum urea and creatinine found to be predictive of hospital mortality in acutely decompensated HF patients [35], did not associate with hospital mortality in our cohort (not shown). A likely explanation for the lack of association is the exclusion of patients with creatinine levels higher than 400 μmol/L and those with severe liver cirrhosis (Child-Pugh B or C). The impact of serum total cholesterol levels on hospital mortality in our cohort was weak and similar to that found in idiopathic cardiomyopathy patients [37], but in contrast to the strong predicting impact of total cholesterol on hospital mortality observed in AHF patients with overlapping renal dysfunction [38] or patients with acute decompensated heart failure [39]. Although low HDL-cholesterol levels were associated with poor prognosis [22] and increased mortality [21] in HF patients, the HDL-cholesterol levels had no impact on the hospital mortality in our cohort. The observed discrepancies between findings in different studies most likely reflect differences in the study populations in terms of sample size, age and gender, inclusion/exclusion criteria, medication or severity and nature of the disease.

To our knowledge, our study is the first to investigate the association of HDL cholesterol efflux capacity with outcome in AHF patients. In a previous study, which compared patients with ischaemic cardiomyopathy and healthy controls, the cholesterol efflux and anti-oxidative capacity of HDL emerged as significant independent risk factors for HF [23]. Based on these findings, one would expect stronger associations of reduced cholesterol efflux capacity and PON-1 AE activity with hospital mortality in our AHF cohort. However, the patients in the previous study [23], in contrast to our patients, were exclusively ischaemic cardiomyopathy patients without clinical signs and symptoms of HF. Additionally, the difference in HDL functionality between patients and healthy control subjects is certainly more pronounced than among patients with a shared pathology.

The decreased cholesterol efflux capacity in patients with stable CAD [28] and the association of decreased cholesterol efflux with incident coronary heart disease (CHD) [29] suggest that the removal of cholesterol from coronary arteries (and probably circulating leukocytes) is an important protective activity of HDL. This assumption that HDL quality outperforms HDL quantity for cardiovascular risk prediction stems from general population studies that focused on the canonical HDL function of promoting cholesterol efflux from macrophages. A similar protective role of HDL–mediated efflux might be operative in ischaemic cardiomyopathy patients, where the cholesterol efflux capacity of HDL was identified as a risk factor for HF [23]. In contrast, however, in our AHF cohort, the HDL-mediated cholesterol efflux might have exhibited its protective capacity in hardly a half (45.4%) of the patients who had evident atherosclerosis. Considering the potency of HDL to remove excess of cholesterol from coronary artery walls [12], a higher HDL efflux capacity might be accompanied by better myocardial perfusion and a concomitantly improved heart performance. Accordingly, it is tempting to speculate that the association of efflux with mortality would be more pronounced in an AHF population with a higher percentage of patients with coronary atherosclerosis. Moreover, HDL-mediated removal of cholesterol from plaques in peripheral arteries may conceivably, by decreasing peripheral resistance, further contribute to spare or improve the function of the failing heart.

A more direct beneficial effect of HDL on the function of the failing heart might be explained by the beneficial effects of HDL on cardiomyocytes, such as attenuation of apoptosis, improved cell survival and preservation of mitochondrial function [40, 41].

The main study strength is its comprehensive design as a prospective single-centre observational and non-interventional study, with a highly structured protocol and predefined statistical analyses. An additional strength is the novel use of HDL functionality as a predictor of hospital mortality in AHF patients. Serum samples were uniformly collected and assays correspondingly performed, which minimised sample preparation bias. The study included well-defined patients, thus allowing a comparison with similar populations. However, the overall sample size was modest and therefore our ability to definitely evaluate the association between cholesterol efflux capacity and AE activity with overall survival was limited. Furthermore, although commonly used, the HDL-cholesterol efflux capacity assay may have some limitations: The assay employs murine macrophages thus excluding the contribution of patients’ macrophages to the overall cholesterol efflux capacity. Moreover, apoB-depleted serum contains in addition to HDL various proteins capable of mediating cholesterol efflux, which may interfere with the contribution of HDL.

## Conclusions

Based on our results obtained by a univariable logistic regression analysis, we conclude that cholesterol efflux capacity, but not AE activity, is associated with hospital mortality in AHF patients. In a multivariable logistic regression analysis no significant association of efflux capacity with hospital mortality was found, suggesting that cholesterol efflux capacity is not an independent risk factor for hospital mortality in AHF patients. However, larger studies are needed to draw firm conclusions.
